# Root Preparation of Deciduous Teeth: Efficacy of WaveOne and ProTaper Systems with and without Passive Ultrasonic Irrigation

**DOI:** 10.22037/iej.v13i3.17094

**Published:** 2018

**Authors:** Bruno Marques da Silva, Fabrício Scaini, Flávia Sens Fagundes Tomazinho, Carla Castiglia Gonzaga, Marilisa Carneiro Leão Gabardo, Flares Baratto-Filho

**Affiliations:** a *School of Health and Biological Sciences, Universidade Positivo, Rua Prof. Pedro Viriato Parigot de Souza 5300, 81280-330, Curitiba, Paraná, Brazil*

**Keywords:** Deciduous Teeth, Endodontic, Passive Ultrasonic Irrigation, Root Canal Preparation, Ultrasonic

## Abstract

**Introduction:**

The aim of this study was to compare root preparation of deciduous teeth with WaveOne Large (WO) and ProTaper F4 (PT) instruments with or without passive ultrasonic irrigation (PUI).

**Methods and Materials:**

Forty-eight deciduous teeth were scanned before and after root preparation and divided in four groups (*n*=12): WO+EDTA (WO); WO+EDTA with PUI (WOPUI); PTF+EDTA (PT); and PT+EDTA with PUI (PTPUI). Root canal enlargement by micro-computed tomography and root canal cleaning by scanning electron microscopy (SEM) were analyzed. Data were submitted to two-way ANOVA and Tukey’s tests to analyze the root canal volume variation, and Kruskal-Wallis followed by Friedman and Wilcoxon tests were used to evaluate the cleaning efficacy. The level of significance was set at 0.05.

**Results:**

No significant difference occurred in total volume between groups (*P*>0.05). On analysis by thirds of the root canal, there was a difference in volume between WO (cervical) compared to WO and PT (apical), and PTPUI (middle and apical) (*P*<0.05). When cleaning of the thirds within the same group was compared, there was a significant difference in all groups (*P*<0.05). Among the groups, in the thirds, in the cervical a difference occurred (*P*=0.028), and the pairwise comparisons indicated statistically difference between WO and PT, and WO and PTPUI (*P*<0.05). In the pairwise comparisons among thirds, in the groups, difrences occured in all of them when compared the cervical and apical thirds (*P*<0.05).

**Conclusion:**

Passive ultrasonic irrigation has not improved the smear layer removal in deciduous teeth. Despite the differences in performance between WO and PT instruments, both were suitable for preparation of deciduous teeth.

## Introduction

The early loss of deciduous teeth, besides being able to alter the sequence and chronology of eruption of permanent teeth, is one of the main causes of malocclusion in permanent dentition [1]. This early loss may be attributed to complications related to endodontic treatment in deciduous teeth element because of dental caries and/or trauma [2]. Therefore, endodontic intervention in deciduous teeth should be fast and simple to enable adequate root canal cleaning without causing weakening of the tooth structure and without risk to the adjacent permanent tooth with the objective of retaining them in the oral cavity until natural exfoliation [3].

The use of rotary instruments in endodontic treatment of deciduous teeth was recommended by Barr *et al.* [4] because it provides adequate root canal cleaning and reduces clinical chair time [5-9]. Endodontic instruments made of nickel titanium (NiTi) that work with reciprocating movement, with a single file to prepare root canals, led to new perspectives in root canal therapy. This concept of using a single instrument for the entire preparation alludes to technique simplification [10, 11]. Studies have reported that reciprocating systems provide quality root canal preparation that is similar to continuous instrumentation with multiple instruments [12, 13].

Root canal preparation is one of the most important steps in endodontic treatment and aims at removing pulp tissue, facilitating the action of the irrigating solution and future filling [14]. Since deciduous teeth have a complex anatomy with thin walls, sharp curvatures and several lateral and accessory canals, the irrigating solution plays a fundamental role in cleaning and disinfecting areas not accessible to endodontic instruments [15]. To optimize the action of irrigation solutions, passive ultrasonic irrigation (PUI) has been used [16-18]. This method is important to remove the smear layer [19], and especially in deciduous teeth with initial clinical signs and symptoms or pulpal necrotic status, it can negatively affect the outcome [20].

**Figure 1:**
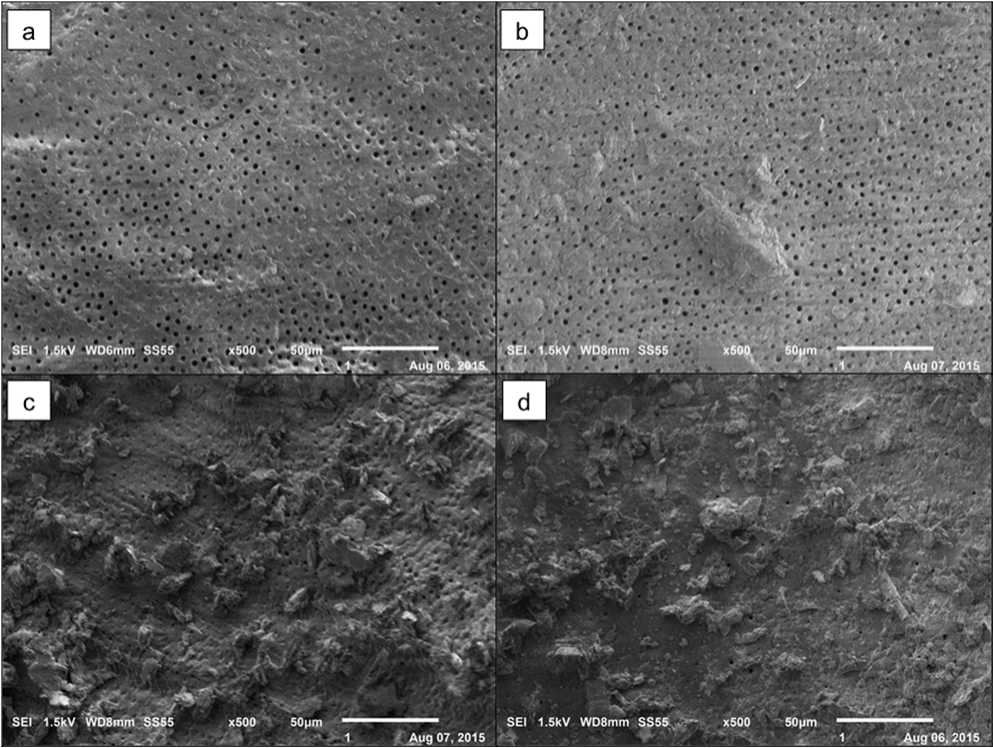
Score used to analyze images in SEM. *A)* Score 1: open dentinal tubules, without debris; *B)* Score 2: open dentinal tubules, with debris covering less than 50% of the area; *C)* Score 3: open dentinal tubules, with debris covering more than 50% of the area; and *D)* Score 4: covered dentinal tubules and debris in 100% of the area examined

Thus, the aim of the study was to compare root canal preparation capacity of WaveOne Large (40/0.08) (WO) (Dentsply Maillefer, Ballaigues, Vaud, Switzerland) and ProTaper F4 (40/0.06) (PT) (Dentsply Maillefer, Ballaigues, Vaud, Switzerland), using a single instrument with and without PUI. The hypotheses tested were that *i)* there is no root preparation difference when using these instruments and *ii)* agitation of the irrigation solution produces a greater cleaning of the root canal wall.

## Materials and Methods

There were 48 uniradicular deciduous teeth with a single canal. Visual and radiographic (mesiodistal and buccolingual) evaluations were done. The teeth selected had no sign of internal or external root resorption. The foraminal patency was verified with a #10 K-file (Dentsply Maillefer, Ballaigues, Vaud, Switzerland) and a #20 FF-file (Dentsply Maillefer, Ballaigues, Vaud, Switzerland) that should enter without resistance to the working length, which was visually set 1 mm shorter than the actual tooth length.

The specimens were mounted on custom devices and were then scanned before and after root preparation in a high-resolution microtomograph at -90 KV and 88 mA (Skyscan 1172; Skyscan, Kontich, Antwerp, Belgium). The obtained cuts had a thickness of 30 μm. The images were captured with Skyscan software (Bruker microCT, Kontich, Antwerp, Belgium) and converted into BMP format.

After pre-preparation scanning, the previous volume of each tooth was calculated for distribution in groups, so that root canals volumes were equivalent. The specimens were initially divided into two groups (*n*=24). Then, the apices were sealed with wax and assembled into a device to simulate the periodontal ligament.

One group had the root canals prepared with the PT (40/0.06) and the other with WO (40/0.08), appropriate to the manufacturer's statement. All instrumentation was performed by the same operator using the X-Smart Plus electric motor (Dentsply Maillefer, Ballaigues, Vaud, Switzerland) per specific program for each instrument. During preparation, irrigation was done with 2 mL of 2.5% sodium hypochlorite at each 3 mm advance of the instrument inside the root canal.

The analysis with micro-computed tomography (micro-CT) allowed the evaluation of root canal enlargement. This was obtained by the difference between total volume before and after the chemical-mechanical preparation. Each root canal was also divided into thirds (cervical, middle and apical) to evaluate each region separately.

The passive ultrasonic irrigation (PUI) was performed with CVDent1000 (CVDentus, São José dos Campos, São Paulo, Brazil) with a T0S-E2 insert at 10% power, 1 mm short of the work length.

After root canal preparation, each group was subdivided (*n*=12) according to the final irrigation protocol into four groups:

WO group: WO+6 mL of 17% EDTA (1 min)+5 mL distilled water; WOPUI group: WO+2 mL of 17% EDTA at each PUI cycle (3 activations of 20 sec)+5 mL of distilled water; PT group: PT+6 mL of 17% EDTA (1 min)+5 mL of distilled water; PTPUI group: PT+2 mL of 17% EDTA at each PUI cycle (3 activations of 20 sec)+5 mL of distilled water.

After post-preparation analysis with micro-CT, two longitudinal grooves were performed throughout the root length on the buccal and lingual walls by a diamond double-face disc of 0.10 mm in thickness and 22 mm in diameter (KG Sorensen, Cotia, São Paulo, Brazil). The resulting grooves reached a depth near the root canal, but without communicating with it. After groove development, the roots were washed in running water to remove debris. With the aid of a chisel, the roots were cleaved into two halves. With a digital caliper (Starrett, Itu, São Paulo, Brazil), the halves were divided into thirds, and then positioned and analyzed using scanning electron microscopy (SEM) (JSM 6010, JEOL, Peabody, Massachusetts, USA) at a power of 20 kV. Magnifications of 500 were used to analyze the presence of smear layer.

The images were digitally recorded, analyzed, and classified into four categories of scores adapted from Kato *et al.* [21], as follows: *score 1*-open dentinal tubules, without debris; *score 2*-open dentinal tubules, with debris covering less than 50% of the area; *score 3*-open dentinal tubules, with debris covering more than 50% of the area; and *score 4*-covered dentinal tubules and debris in 100% of the area examined (Figure 1).

Images analysis was performed by two independent examiners, previously calibrated (kappa=0.83), blind to the study and following pre-established criteria.

The root canal volume variation, considering the methods of preparation in the thirds, was evaluated using the two-way ANOVA and Tukey’s test. The cleaning efficacy score among groups and third was analyzed using the Kruskal-Wallis. The Friedman test was used to verify the difference among thirds within the same group, and the Wilcoxon test verified if, within the same group, there was difference in the pairwise comparisons between thirds. The analysis was performed with the Statistical Package for the Social Sciences (SPSS22.0, SPSS Inc., Chicago, Illinois, USA). The level of significance was set at 0.05. 

## Results

There was no fracture of any instrument, formation of zip, steps, or perforation of root canals. In the comparison of total volume variation, there was no statistically significant difference among groups (*P*>0.05). The difference occurred among the cervical third of the WO was compared to the apical thirds of WO and PT, and middle and apical thirds of PTPUI (*P*<0.05) (Table 1).

**Figure 2:**
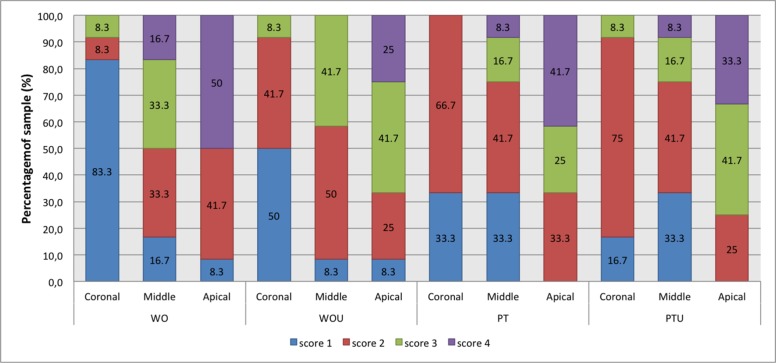
Score distribution of smear layer (in %) in the cervical, middle and apical thirds in the groups WO, WOPUI, PT, and PTPUI

The root canal cleaning ability was evaluated by the presence of smear layer verified by SEM (Table 2). The distribution of obtained scores in each group and region is shown in Figure 2.

Among the groups, in the thirds, in the cervical a difference occurred (*P*=0.028), and the pairwise comparisons indicated statistically difference between WO and PT, and WO and PTPUI (*P*<0.05). In the pairwise comparisons among thirds, within the groups, differences occured in all of them when compared the cervical and apical thirds (*P*<0.05).

## Discussion

This study advocates the use of a single instrument, both in reciprocating (WaveOne Large) and rotational (ProTaper F4) motion, to compare and establish a better protocol of attendance and to expedite care of the pediatric patient.

The use of rotatory systems in deciduous teeth has been considered safe, fast and efficient [3-5, 7, 9], with better cutting efficiency [9, 22] shaping with less straightening, and more centered preparations of curved primary root canals [23]. Then, the concept of root canal preparation with a single instrument was advocated in 2008 [10] and was quickly assimilated by endodontists worldwide. It is a more efficient and faster technique in relation to the use of multiple instruments [11]. In the care of children, this is important because it reduces the stress of the patient who cannot stay in the chair for a long time [6, 8]. Regarding the materials and methods employed here, the samples were analyzed by micro-CT before and after root canal preparation and later analyzed by SEM for assessing the cleaning of root canal. Micro-CT has been used for this purpose [24, 25] because it has the advantage of not being invasive and not destroying the samples for confirmation of results.

In the present study, samples were allocated so that groups had root canals with equivalent volume. For this, a pre-preparation scan was performed to calculate the root canal volume of each tooth. This allowed a homogeneous distribution between groups, since canines and incisors with single channel, but different internal anatomies, were used. 

**Table 1:** Mean (SD) of volume change per root canal third and total volume in mm^3^

**Group**	**Root canal third**				
	**Cervical**	**Middle**	**Apical **	**Total **
**WO**	2.05 (1.74)^a^	1.28 (0.89)^ab^	0.75 (0.35)^b^	4.08 (2.89)
**WOPUI**	1.69 (0.96)^ab^	1.21 (0.74)^ab^	0.85 (0.46)^ab^	3.74 (2.02)
**PT**	1.80 (1.57)^ab^	1.05 (0.82)^ab^	0.70 (0.42)^b^	3.55 (2.71)
**PTPUI**	1.43 (1.16)^ab^	0.63 (0.26)^b^	0.57 (0.22)^b^	2.63 (1.52)

*Two-way ANOVA and Tukey test. Different letters indicate statistically significant differences (P<0.05)*

**Table 2:** Median and interquartile range (IR) debris removal scores in the root thirds by SEM

**Group**	**Root canal third**		
	**Cervical**	**Middle**	**Apical**
**WO**	1.00 (0.00)^A,b^	2.50 (1.00)^a^	3.00 (2.00)^a^
**WOPUI**	1.50 (1.00)^A,C,b^	2.00 (1.00)^a^	3.00 (1.75)^a^
**PT**	2.00 (0.00)^B,C,a^	2.00 (1.75)^a^	3.00 (1.75)^b^
**PTPUI**	2.00 (1.00)^B,C,a^	2.00 (1.75)^a^	3.00 (2.00)^b^
***P*** **-value** *	0.028	0.374	0.920

* *Kruskal-Wallis test. Bold values are statically significant (P<0.05); Note: Different letters indicate statistically significant differences (P<0.05). Capital letters indicate the pairwise comparisons among groups in the cervical third, Friedman test. Median with different superscript lowercase letters are statistically different within each row according to the Wilcoxon test*

The results showed no statistically significant difference in the volume variation between systems in the cervical, middle, and apical thirds. This was expected since the WO and PT are, in general, very similar in their shape and conicity. Although not significant, difference was found in the apical third between WO and PT, regardless of the use of ultrasound during irrigation. This can be explained by the instrument design, since in the apical millimeters of WO large, the taper is 0.08, while for PT F4, the taper is 0.06; thus, the conicity of the preparations of the former is greater than that of the latter instrument. Ultrasound has been shown to be useful in several stages of endodontic treatment, for example, in cleaning the root canals [26, 27]. The methods of activated irrigations showed significantly better smear layer scores compared to manual irrigation [28], but there is a scarcity in the research literature with deciduous teeth.

In this study, in relation to cleaning, the only statistical difference occurred in the cervical third, between WO and PT/PTPUI groups. This result may be related to greater enlargement obtained in this region in the WO group, due to the movement of brushing performed, as recommended by the manufacturer.

Katge *et al.* [6] analyzed 120 root canals of primary molars prepared with WO, PT and manual files. Their results indicated that WO was more effective in the coronal and middle thirds of the root canal, when compared to the other methods. But, at the apical level, no statistical difference between the three systems occurred.

Regarding the removal of smear layer authors indicated that in deciduous teeth with symptomatic or necrotic pulp, the remnants can negatively affect the outcome [20]. The use of PUI is an important method with this aim [19]. Furthermore, has been used currently in endodontic treatment and considered safe [29]. Regarding the removal of the intracanal smear layer, and evaluation of different endodontic irrigation and activation systems, including the PUI, showed that complete removal is not possible, with worse results in the apical third [30]. Differently from the literature [26, 31], considering the methodological differences in the present study, it was verified that in groups where the ultrasound was used, there was no improvement in performance. The analysis by micro-CT showed that in groups where there was complementation with the use of ultrasound, the volume variation was smaller than in groups without it. This may be justified because the tip of the device touches root canal walls, which causes more debris [21, 32] and erosion [33]. Nonetheless, the literature recommends the instrument to vibrate freely within the root canal to promote cleanliness [32].

Further studies are required to evaluate the effectiveness of mechanized instrumentation of the root canals of deciduous teeth and to validate irrigation solution agitation methods to complement the removal of smear layer after preparation.

## Conclusion

Passive ultrasonic irrigation has not improved the smear layer removal in deciduous teeth. Despite the differences in performance between WO and PT instruments, both were suitable for preparation of deciduous teeth.

## Conflict of Interest:

‘None declared’.
